# Serotonin stimulated parathyroid hormone related protein induction in the mammary epithelia by transglutaminase-dependent serotonylation

**DOI:** 10.1371/journal.pone.0241192

**Published:** 2020-10-23

**Authors:** Celeste M. Sheftel, Laura L. Hernandez

**Affiliations:** 1 Molecular and Cellular Pharmacology Training Program, University of Wisconsin-Madison, Madison, Wisconsin, United States of America; 2 Department of Animal and Dairy Sciences, University of Wisconsin-Madison, Madison, Wisconsin, United States of America; Wayne State University, UNITED STATES

## Abstract

Mammary-derived serotonin has been implicated in breast-to-bone communication during lactation by increasing parathyroid hormone related-protein (PTHrP) in the mammary gland. It is well established that PTHrP acts on the bone to liberate calcium for milk synthesis during lactation; however, the mechanism of serotonin’s regulation of PTHrP has not been fully elucidated. Recently, serotonylation has been shown to be involved in a variety of physiological processes mediated by serotonin. Therefore, we investigated whether serotonylation is involved in serotonin’s regulation of PTHrP in the mammary gland using lactogenically differentiated mouse mammary epithelial cells. We investigated the effect of increased intracellular serotonin using the antidepressant fluoxetine or 5-hydroxytryptophan (serotonin precursor), with or without transglutaminase inhibition and the corresponding action on PTHrP induction and activity. Treatment with fluoxetine or 5-hydroxytryptophan significantly increased intracellular serotonin concentrations and subsequently increased PTHrP gene expression, which was reduced with transglutaminase inhibition. Furthermore, we determined that transglutaminase activity is increased with lactogenic differentiation and 5-hydroxytryptophan or fluoxetine treatment. We investigated whether RhoA, Rac1, and Rab4 were potential serotonylation target proteins. We speculate that RhoA is potentially a serotonylation target protein. Our data suggest that serotonin regulates PTHrP induction in part through the process of serotonylation under lactogenic conditions in mouse mammary epithelial cells.

## Introduction

Serotonin (5-hydroxytryptamine (5HT)), an established monoamine and neurotransmitter, is synthesized from L-tryptophan in a 2-step conversion. First, tryptophan hydroxylase (TPH), the rate-limiting enzyme, hydroxylates L-tryptophan producing 5-hydroxytryptophan (5HTP), which is then decarboxylated by aromatic L-amino acid decarboxylase producing 5HT [1]. While 5HT is commonly known as a central neurotransmitter altering behavior and mood, over 95% of 5HT is produced in the periphery largely in the gut enterochromaffin cells in a non-lactating state. However, during lactation, it has been has demonstrated that the mammary gland contributes approximately 50% of circulating 5HT [2, 3]. 5HT is then released into the blood and stored by platelets, which lack the TPH1 enzyme. Degradation of 5HT occurs through oxidation by monoamine oxidase (MAO) into 5-hydroxyindolacetic acid which is excreted in the urine [4, 5]. In humans and in mice, there are two TPH enzymes: TPH2 which converts L-tryptophan to 5HTP in the central nervous system and TPH1 which converts L-tryptophan to 5HTP in the periphery [6–8]. 5HT and TPH1/2 are unable to cross the blood brain barrier, resulting in distinctly separate compartmentalization of 5HT [6, 9].

Mammary-derived 5HT is important in regulating maternal calcium homeostasis and breast-to-bone communication via the synthesis and secretion of the parathyroid hormone related protein (PTHrP) in the mammary gland during lactation [10–12]. PTHrP is secreted from the mammary epithelial cells and then acts on the bone to liberate calcium for milk synthesis [13–15]. Since the fetus is not fully mineralized in utero, there is a disproportionate demand for calcium post-partum to be excreted into milk to support the neonate. This results in utilization of the maternal bone to supply a large portion of the calcium for the infant through the milk, resulting in up to 10% maternal bone loss during the recommended 6 months of exclusive breastfeeding [16–19]. Additional bone stressors on the mother, such as the use of antidepressants like selective serotonin reuptake inhibitors (SSRIs) have been demonstrated to result in a sustained reduction in trabecular bone density [20, 21]. Furthermore, our lab has shown *in vivo* peripartal exposure to the SSRI, fluoxetine (FLX), leads to an increase in mammary gland 5HT content and consequently PTHrP (*Pthlh* gene) during lactation, with a sustained reduction in maternal trabecular bone [22].

SSRIs are the first-choice treatment for peripartum depression due to low fetal teratogenicity and high patient compliance; often the benefits of the SSRI outweigh the potential negative effects of use [23–27]. SSRI’s exert their action by blocking the 5HT reuptake transporter, SERT, which results in increasing exposure of a neuron or tissue to 5HT. This secondarily upregulates TPH1/2, increasing 5HT synthesis while also decreasing 5HT degradation [28]. We have previously demonstrated an epigenetic-hedgehog link between 5HT and PTHrP involving DNA methylation [29]. However, 5HT has no donatable methyl groups, suggesting a secondary mechanism or indirect regulation of DNA methylation.

Novel research in the past decade has uncovered a covalent post-translational modification involving 5HT, termed serotonylation [30]. In this reaction, 5HT is transamidated onto a glutamine residue of a target protein via the calcium dependent enzyme, transglutaminase (TG) [31]. In humans there are 9 TG genes, resulting in eight active enzymes; however, TG2 (termed tissue transglutaminase) is ubiquitously expressed and generally considered to be the most-likely TG enzyme involved in the transamidation of monoamines [31, 32]. Serotonylation was first described in platelet α-granule release through the activation of small GTPases [30]. Since then, it has been implicated in vascular processes (i.e., smooth muscle contraction), pulmonary hypertension, glucose metabolism, dendritic spine remodeling in the brain, and most recently in histone modifications altering transcription [33–38]. Small G-proteins such as Rho, Rab4, or Rac1 are common target proteins for serotonylation [30, 36, 39, 40].

TG2, the enzyme responsible for serotonylation, has been implicated in the promotion of growth, survival, and metastasis of breast cancer in mammary epithelial cells, however the molecular process of serotonylation has not been explored in normal lactogenic mammary epithelial cells [41, 42]. Given the possible epigenetic effects of 5HT on PTHrP in the lactogenic mammary gland, and the lack of donatable methyl groups on 5HT, investigation of the possibility of serotonylation in the regulation of this pathway seemed prudent. Additionally, there has been no focus on the role of serotonylation during the normal lactogenic processes.

Herein, we examined the role of TG2 in the mechanism involving 5HT’s regulation of PTHrP under lactogenic conditions. We hypothesized *in vitro* lactogenic differentiated mammary epithelial cells pharmacologically manipulated to increase intracellular 5HT, will see a corresponding increase in PTHrP expression via the molecular process, serotonylation. Furthermore, we investigated the role of a potential serotonylation target protein, the small G protein RhoA.

## Materials and methods

### Cell culture

Mouse mammary epithelial cells (HC11), a prolactin responsive cell line that undergoes lactogenic differentiation, were utilized for this experiment [43]. HC11 cells were plated at a seeding density of 250,000 cells/well in a 12 well plate or 1,000,000 cells/10cm plate, sustained in proliferation media (RPMI 1640, 10% FBS, 1% antibiotics, 5μg/mL insulin, 25ng/mL EGF) and 48 hours later were confluent. Once confluent, EGF was then removed (proliferation media: RPMI 1640, 10% FBS, 1% antibiotics, 5μg/mL insulin) for 48 hours to initiate lactogenic differentiation. After 48 hours, lactogenic media (RPMI 1640, 10% FBS, 1% antibiotics, 10μg/mL insulin, 1μg/mL prolactin, 0.5μg/mL hydrocortisone) was then added to induce lactogenic differentiation. The following treatments were then administered as following: 40μM FLX (Sigma Aldrich, #F132, St. Louis, MO), 500 μM 5HTP (Sigma Aldrich, #H9772, St. Louis, MO), 25μM (mono)dansylcadaverine (MDC) dissolved in DMSO (Sigma Aldrich, #30432, St. Louis, MO), or DMSO (control) was added to the lactogenic media. After 48 hours of treatment, cells were harvested. Cell culture differentiations were done in duplicate and was replicated three times.

### RNA and RTqPCR

HC11 cells were harvested with TRI-Reagent (Molecular Research, Thermo Fisher Scientific, #NC9277980, Waltham, MA) according to manufacturer’s protocol. 1μg RNA was reverse transcribed with High-Capacity cDNA Reverse Transcription Kit (Applied Biosystems, Thermo Fisher Scientific, #4368814, Waltham, MA) with murine RNase inhibitor (New England Biolabs, #M0314L, Ipswich, MA). Quantitative RT-PCR was conducted using the CFX96 Touch-Real-Time PCR Detection System (Bio-Rad Laboratories, Hercules, CA). Reaction mixtures and cycling conditions were performed as previously described [44]. Primers were designed with an optimal annealing temperature of 60°C. Amplification efficiencies of primers were accepted within a range of 95–105% and a singular melt-curve. The primer sequences are listed in Table 1. The housekeeping parameter was the geometric mean of *Rsp9* and *S15*. Analysis was conducted using the 2^-ΔΔCT^ method.

**Table 1:** Primer sequences for the studied genes quantified by real-time-PCR.

Genes	Forward Primer (5’—3’)	Reverse Primer (5’—3’)
*rpS15*	TTGAGAAAGGCCAAAAAGGA	GTTGAAGGTCTTGCCGTTGT
*rpRSP9*	GGAGACCCTTCGAGAAGTCG	GGGGATCCTTCTCGTCTAGC
*Pthlh*	TTCCTGCTCAGCTACTCCGT	GATGGACTTGCCCTTGTCAT
*WAP*	TATCATCTGCCAAACCAACG	TAGATTCCAAGGGCAGAAGC
*TG2*	GATCCTCGCTTGAGTGTCCC	TTCTCTTGGCATAGGTCGGC
*MAO-A*	ACAGCAACACAGTGGAGTGG	GGAACATCCTTGGACTCAGG
*RhoA*	CTGTCGGGAGTTGGACTAGC	CAGTTTCTTCCTGATGGCAGC
*SERT*	ATCACGCTGGGTTTGGATAG	ATGACCACGATGAGCACAAA
*Tph1*	CCCGGAAATCAAAGCAAAG	CTTCCTTCGCAGTGAGCTG
*Rab4a*	CAGCCGAGAAACCTACAATGC	TCCAAGTCCTTCTTGTTCCCG
*Rab4b*	ACTATCGGCGTGGAGTTTGG	CCCCTCGGTAATAACTCCGC
*Gli1*	**GGCAGGGAAGAGAGCAGACT**	**ACTGCCTGCTGGGGAGTG**

Primers were designed using Primer 3. All primers were run at an annealing temperature of 60°C and the geometric mean of the ribosomal protein *S15* and ribosomal protein *RSP9* were used as the housekeeping gene.

Abbreviations: rpS15, ribosomal protein S15; rpRSP9, ribosomal protein RSP9; Pthlh, parathyroid hormone related protein; WAP, whey acidic protein; TG2, transglutaminase 2; MAO-A, monoamine oxidase A; SERT, serotonin reuptake transporter; Tph1, tryptophan hydroxylase 1.

### Protein extraction and immunoblotting

HC11 cells were harvested with radioimmunoprecipitation assay buffer (1X PBS, 1% nonidet P-40, 0.5% sodium deoxycholate, 0.1% SDS) supplemented with 10μL/mL Halt Protease and Phosphatase Inhibitor Cocktail (Thermo Fisher Scientific, #78441, Waltham, MA). Lysates were homogenized and cleared with centrifugation for 15 minutes at 12,000xG. Protein concentration was determined using bicinchoninic acid assay (Bioworld, #20831001–1, Dublin, OH). Protein lysates were diluted to 1.5μg/μl with 5x sample buffer containing SDS and β-mercaptoethanol and heated at 95°C for 10 minutes. 15μg protein was separated by electrophoresis on a gradient (8–20%) SDS-polyacrylamide gel and transferred for 1 hour at 100V onto a polyvinylidene difluoride membrane (Millipore Sigma, #IPVH00010, Burlington, MA). Membranes were blocked for 1 hour with Sea Block Blocking Solution (Thermo Fisher Scientific, Waltham, MA) and probed overnight at 4°C with 1:1000 rabbit polyclonal TG2 (Abcam, #ab421, Cambridge, United Kingdom) and 1:1000 rabbit polyclonal β-actin (Cell Signaling Technology, #4967S, Danvers, MA). The following day, the membrane was washed 3 times with TBST and probed 1:5000 with fluorescent secondary antibodies (Li-Cor Biosciences, IRDye 800 CW #925–32213, IRDye 680 RD #925–68070, Lincoln, NE), then washed 3 times with TBST. Protein bands were detected using Li-Cor Odyssey Fc (Li-Cor, Lincoln, NE) with a 2-minute exposure for 700 channel and 10-minute exposure for 800 channels. Image analysis and protein band quantification were performed using Image Studio Lite software (Li-Cor Biosciences, version 5.2, Lincoln, NE).

### Assays

Intracellular 5HT concentrations were determined using a Serotonin Enzyme Immunoassay Kit (Beckman Coulter, #IM1749, Brea, CA) using 50μg total protein as we have previously described [12, 22]. Intracellular cyclic adenosine monophosphate (cAMP) concentrations were determined using a cyclic AMP XP Assay Kit (Cell Signaling Technology, #4339S, Danvers, MA) using 50μg total protein per the manufacturer’s instructions. Intracellular transglutaminase activity was determined using a Transglutaminase Assay Kit (Sigma-Aldrich, #CS1070, St. Louis, MO) using 20μg total protein per manufacturer’s instructions. Intracellular RhoA activity was determined using RhoA G-LISA Activation Assay Kit (Cytoskeleton, #BK124, Denver, CO) using 20μg total protein per the manufacturer’s instructions. Intracellular Rac1 activity was determined using a Rac1 G-LISA Activation Assay Kit (Cytoskeleton, #BK128, Denver, CO) using 25μg total protein per the manufacturer’s instructions. All assays had an intra-assay CV of <10% and the inter-assay CV of <5%.

### Statistical analysis

All statistical analyses were conducted using Graph Pad Prism 8 (Version 8.4.0). Analysis between two treatments was performed using a Student’s unpaired two-sided *t* test and analysis between multiple treatments were performed using one-way ANOVA. Bartlett’s test was applied to test equal variances among treatments. If sample populations did not have equal variances, Welch correction for unequal variance was applied. Analyses with multiple time points were conducted using a two-way ANOVA with repeated measures. Tukey’s multiple comparisons test was performed to detect differences between treatment groups. For all analyses, differences between the mean were considered significant when *p*<0.05. All values are reported as means ± SEM.

## Results

### HC11 cells undergo lactogenic differentiation and transglutaminase protein and activity are observed

**Fig 1:**
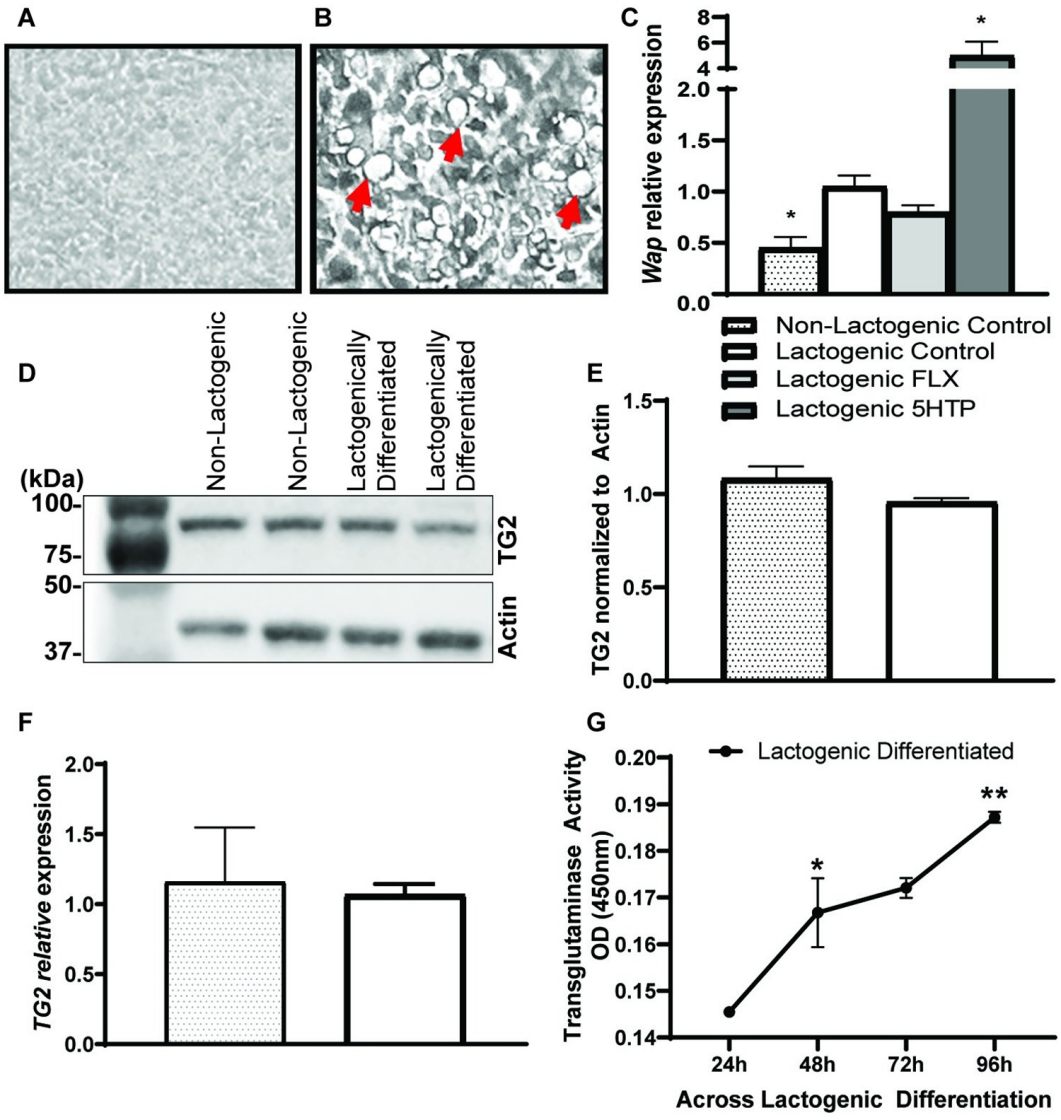
Transglutaminase protein and activity are observed in mouse mammary epithelial cells. **A.** Brightfield image of non-lactogenic HC11 cells, not treated with prolactin or hydrocortisone **B.,** and lactogenically differentiated HC11 cells show morphological changes in the form of mammosphere production seen by the arrows. **C.** Whey acidic protein (*Wap*) gene expression is upregulated when treated with lactogenic hormones and further increased with lactogenic hormones + 5HTP. **D**. Western blot analysis of transglutaminase 2 (TG2) in lysate of HC11 cells before differentiation (proliferation media) and after differentiation (lactogenic media), quantified in **E**., show no change in protein expression. TG2 quantification relative to the actin control. **F.** *TG2* gene expression is unchanged before and after differentiation. **G.** TG activity significantly increases overtime in the lactogenic differentiated cells. Values are means and bars indicate SEM (n = 3 independent cell cultures). *p<0.05 **p<0.01 ***p<0.001 ****p<0.0001.

HC11 cells undergo lactogenic differentiation when treated with lactogenic hormones (prolactin, insulin, and hydrocortisone). We confirmed a successful lactogenic differentiation through visualization of mammospheres (Fig 1A and 1B) and upregulation of the milk protein gene, whey acidic protein (WAP) when treated with lactogenic hormones. *Wap* expression was further increased with 5HTP treatment (Fig 1C). We analyzed TG2 protein expression in both undifferentiated and lactogenically differentiated HC11 cells, detecting a presence of TG2, but unchanged when treated with lactogenic hormones (Fig 1D) quantified in Fig 1E. This is further supported by *TG2* gene expression (Fig 1F), which is not affected by lactogenic differentiation. In addition to the presence of TG2 expression at both the mRNA and protein levels in the lactogenically differentiated HC11 cells, TG is active. Using a TG activity assay (Fig 1G), it was determined that TG activity increases after treatment with lactogenic hormones at 48 hours (*p* = 0.0194) and 96 hours (*p* = 0.0022) compared to baseline. Combined, these data establish the cells successfully undergo lactogenic differentiation and the enzyme required for serotonylation is present and active in mammary epithelial cells. Furthermore, activity increases after treatment with lactogenic hormones, suggesting the molecular process of serotonylation can occur during lactation in these cells.

### FLX and 5HTP increase intracellular 5HT concentrations resulting in increased synthesis of PTHrP

**Fig 2:**
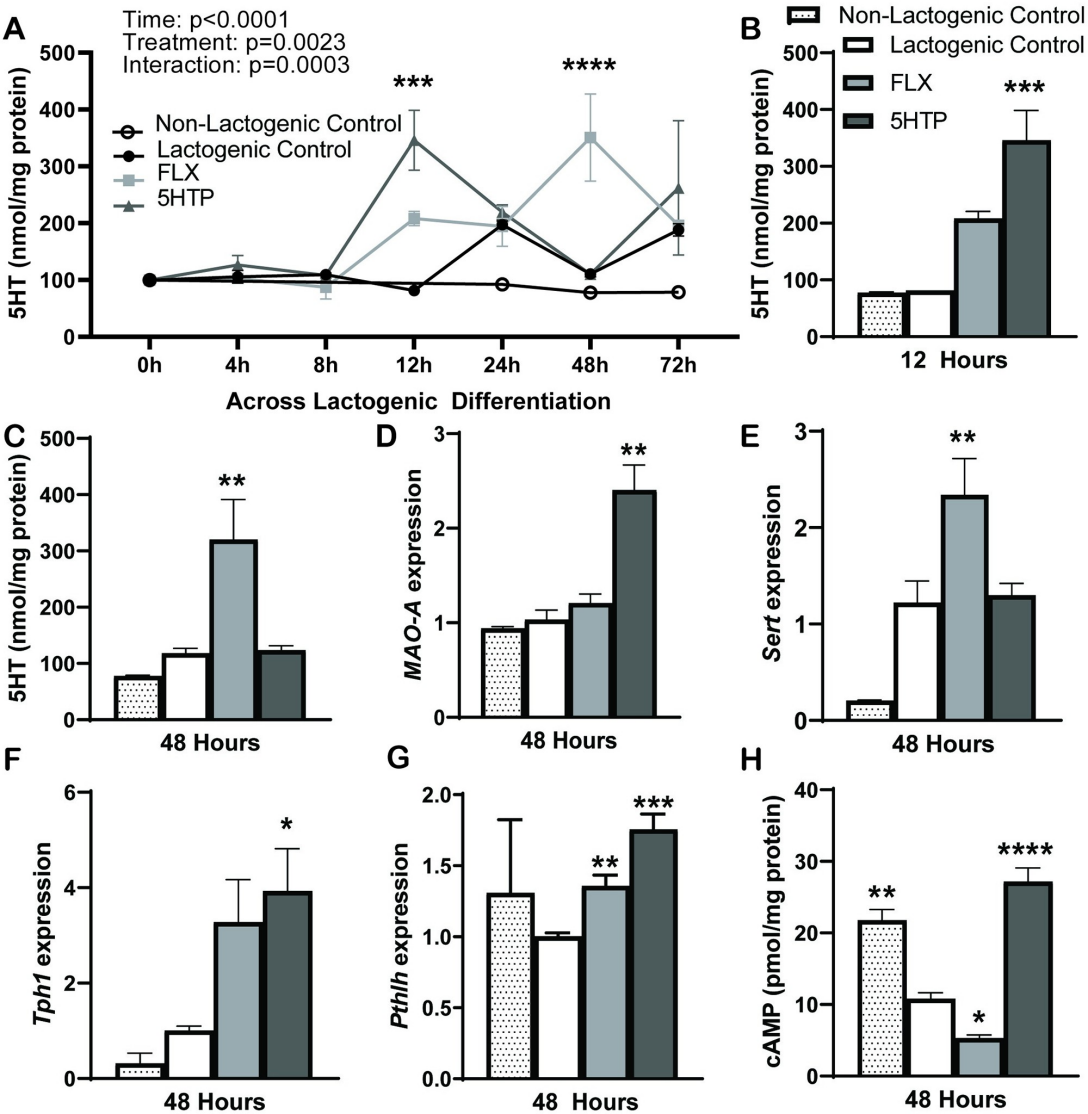
HC11 cells increase Pthlh gene expression and activity via cAMP with increases in intracellular 5HT. **A-C.** 5HTP significantly increases intracellular 5HT at 12- and 72 hours post treatment compared to baseline and at 12 hours compared to lactogenic control **(B).** FLX significantly increases 5HT concentration at 48 hours post treatment compared to baseline and compared to control **(C).** MDC does not impact 5HT concentration (not shown). **D-G.** Gene expression at 48 hours post treatment. **D.** Monoamine oxidase-A (MAO-A) gene expression is significantly upregulated in 5HTP treatment. **E.** *Sert* gene expression in HC11 cells is upregulated with FLX treatment. **F.** *Tph1* gene expression is upregulated with FLX and 5HTP treatment. **G.** *Pthlh* mRNA expression is upregulated with FLX and 5HTP treatment. **H.** cAMP concentration at 48 hours increased in non-lactogenic control cells and 5HTP treated cells, whereas FLX treated cells decreased cAMP concentration compared to lactogenic control. Values are means and bars indicate SEM (n = 3 independent cell cultures). *p<0.05 **p<0.01 ***p<0.001 ****p<0.0001.

Fig 2A depicts 5HT concentrations are affected by time (*p*<0.0001), treatment (*p* = 0.0023), and an interaction of treatment by time (*p* = 0.0003) in response to FLX and 5HTP. 5HT concentrations in 5HTP treated cells peaked at 12- and 72 hours relative to its baseline and at 12 hours compared to lactogenic controls (*p* = 0.0002, *p* = 0.0232, and *p* = 0.0005, respectively) (Fig 2B), whereas FLX treated cells had highest 5HT concentrations 48 hours relative to baseline and compared to lactogenic control (*p*<0.0001) (Fig 2C). We demonstrated a significant upregulation of *MAO-*A gene expression (Fig 2D) in 5HTP treated cells (*p* = 0.0037), suggesting increased degradation of 5HT at 48 hours post treatment. To verify the impact of our treatments on the 5HT pathway, we measured expression of SERT (Fig 2E) and TPH1 (Fig 2F) at 48 hours post treatment. We detected increased expression of *Sert* and *Tph1* when HC11 cells were treated with FLX (*p* = 0.0096 and *p* = 0.0702, respectively). Interestingly, 5HTP treated cells only had increased *Tph1* mRNA expression (*p* = 0.0462). This confirms that only FLX treatment inhibits SERT and both FLX and 5HTP upregulate *de novo* synthesis of 5HT.

Furthermore, we examined the role of PTHrP expression and activity using *Pthlh* gene expression and cAMP concentrations, a common measure of PTH and PTHrP activity. At 48 hours post treatment, *Pthlh* mRNA expression (Fig 2G) was significantly upregulated in FLX and 5HTP treated cells (*p* = 0.0050 and *p* = 0.0005, respectively). At 48 hours post differentiation, 5HTP treated cells significantly upregulated cAMP concentrations (*p*<0.0001) compared to lactogenic control, whereas FLX significantly decreased cAMP concentrations (*p* = 0.0372) compared to lactogenic control (Fig 2H). This suggests in the 5HTP treated cells, *Pthlh* expression and activity is acutely increased. Interestingly, in the non-lactogenic control we a similar expression level of *Pthlh* gene expression and a significant increase in cAMP concentrations (*p* = 0.0015) compared to the lactogenic control.

### Inhibition of TG2 restores *Pthlh* gene expression to lactogenic control levels in HC11 cells

**Fig 3:**
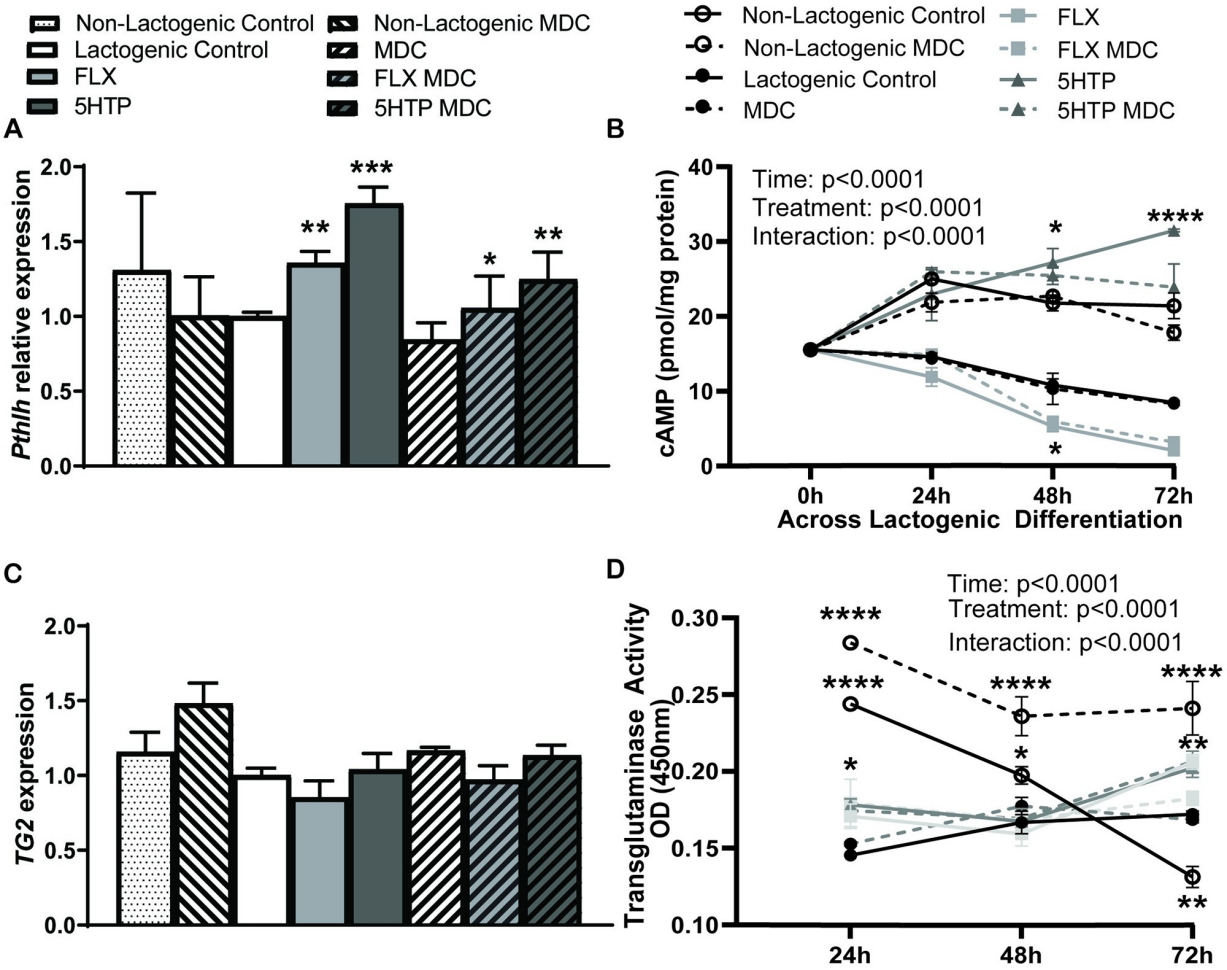
Inhibition of transglutaminase restores *Pthlh* gene expression in HC11 cells. **A.** *Pthlh* mRNA expression is significantly increased in FLX and 5HTP compared to lactogenic control and is significantly decreased in FLX or 5HTP + transglutaminase inhibition (MDC) restoring *Pthlh* mRNA to lactogenic control levels. *Pthlh* mRNA remained unchanged with the controls with MDC treatment. **B.** cAMP concentration is unchanged with transglutaminase inhibition. 5HTP significantly increases at 24-, 48-, and 72 hours compared to baseline. 5HTP is significantly increased at 48- and 72 hours and FLX is decreased at 48 hours compared to lactogenic control. **C.** *TG2* mRNA expression is not changed with treatment. **D.** Lactogenic control increases TG activity between baseline and 72 hours, FLX increases TG activity between baseline and 72 hours as well as between 48 and 72 hours and 5HTP increases TG activity between 48 and 72 hours. MDC treatment kept TG activity constant with no significant changes, except in the non-lactogenic control where MDC treatment had increased TG activity. The non-lactogenic control decreased expression between 24 and 48 hours as well as between 48 and 72 hours. 5HTP was significantly increased at 24 hours compared to lactogenic control and 5HTP and FLX were significantly increased at 72 hours compared to lactogenic control. The non-lactogenic control had increased activity at 24 and 48 hours, but decreased activity at 72 hours compared to the lactogenic control. Values are means and bars indicate SEM (n = 3 independent cell cultures). *p<0.05 **p<0.01 ***p<0.001 ****p<0.0001.

We examined the role of TG2 on induction of PTHrP using a commonly used small molecule inhibitor, MDC, to decrease serotonylation. *Pthlh* mRNA expression was significantly decreased to lactogenic control levels with TG inhibition using FLX or 5HTP combined with MDC (*p* = 0.0247 and *p* = 0.0053, respectively) (Fig 3A). This result suggests TG2 is involved in 5HT’s regulation of *Pthlh* in lactogenic conditions. The non-lactogenic control and lactogenic control remained unchanged with MDC treatment.

Next, we analyzed cAMP concentrations over time and observed significant changes across time (*p*<0.0001), due to treatment (*p*<0.0001), as well as a treatment by time interaction (*p*<0.0001). 5HTP significantly upregulated cAMP concentrations at 24-, 48-, and 72 hours compared to lactogenic control (*p* = 0.0029, *p*<0.0001 and *p*<0.0001, respectively), whereas FLX treated cells decreased cAMP concentrations at 48 hours (*p* = 0.0372). The lactogenic control had significantly decreased cAMP concentrations by 72 hours compared to its baseline (*p* = 0.0026), with FLX decreasing cAMP at 48 and 72 hours relative to its baseline (*p*<0.0001). 5HTP treatment significantly increased cAMP at 24-, 48-, and 72 hours compared to its baseline (*p* = 0.0014, *p*<0.0001, and *p*<0.0001 respectively). The non-lactogenic control also had significantly increased cAMP concentrations compared to the lactogenic control at 24-, 48-, and 72 hours (*p* = 0.006, *p*<0.0001, *p*<0.0001, respectively) and at 24-, 48-, and 72 hours compared to its baseline (*p* = 0.0260, *p* = 0.0287, *p* = 0.0439). Additionally, cAMP concentrations were unchanged with TG inhibition.

TG2 gene expression was unchanged due to treatment (Fig 3C). TG activity (Fig 3D) significantly changed overtime (*p*<0.0001), due to treatment (*p* = 0.0097), and there was a treatment by time interaction (*p* = 0<0.0001). 5HTP significantly increased TG activity at 24 hours (p = 0.0120) and 5HTP and FLX significantly increased TG activity at 72 hours (*p* = 0.0243 an *p* = 0.0084, respectively) compared to the lactogenic control. Interestingly the non-lactogenic control had significantly increased TG activity at 24 and 48 hours (*p*<0.0001 and *p* = 0.0339), however by 72 hours it was significantly decreased (*p* = 0.0042) compared to lactogenic control. The lactogenic control had increased TG activity at 72 hours relative to baseline (*p* = 0.0283). FLX treatment increased TG activity at 72 hours compared to baseline levels (*p* = 0.0027), as well at between 48 and 72 hours (*p*<0.0001), and 5HTP treatment increased TG activity between 48 and 72 hours (*p* = 0.0015). MDC treatment did not result in changes (*p*>0.05) when cells were treated with FLX or 5HTP. The non-lactogenic control significantly decreased TG activity at 24-, 48-, and 72 hours (*p* = 0.0012, *p*<0.0001, and *p*<0.0001, respectively).

### RhoA gene expression is upregulated when intracellular 5HT is increased and RhoA activity decreases over time

**Fig 4:**
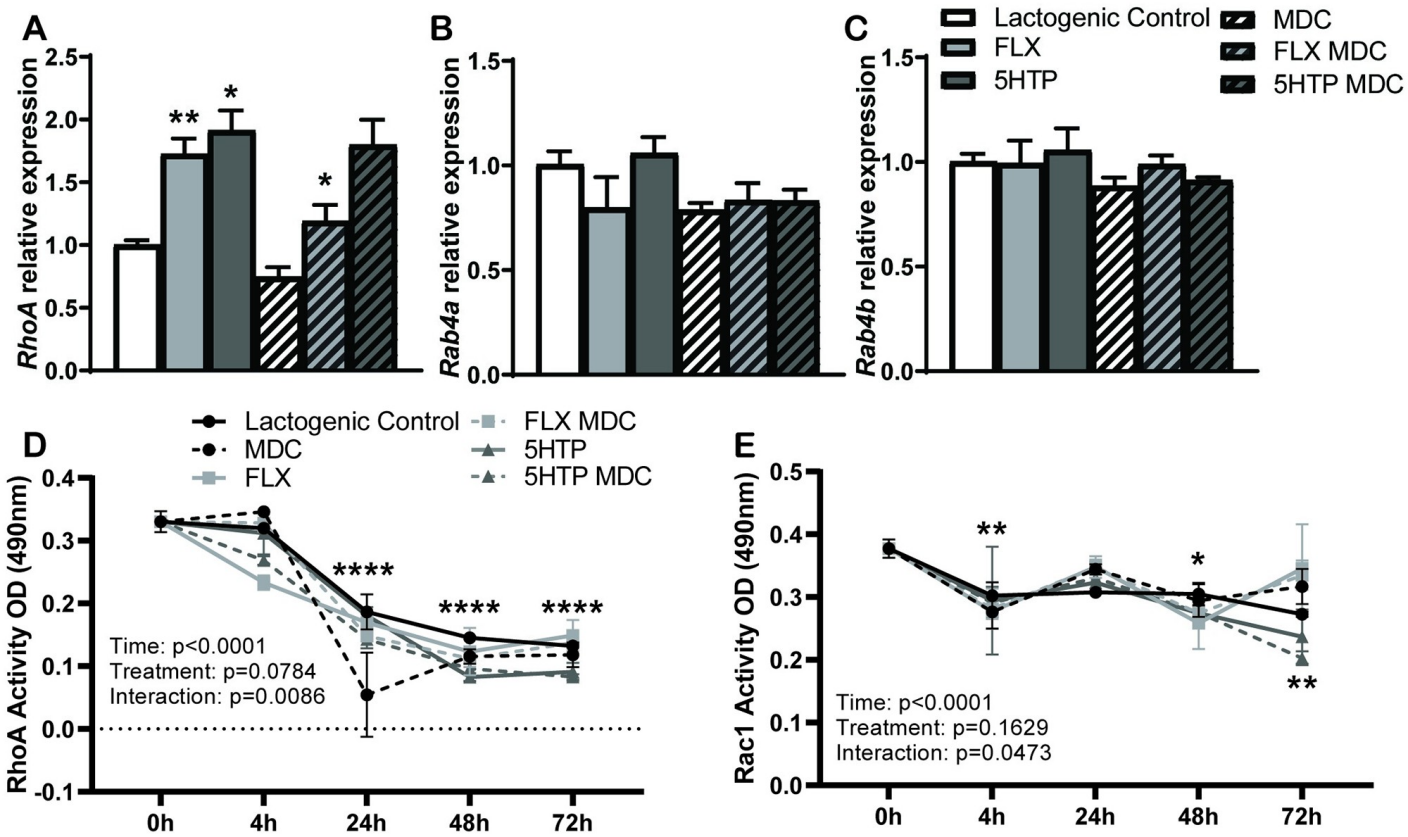
RhoA gene expression and activity are impacted with increased intracellular 5HT. **A.** *RhoA* mRNA expression is significantly upregulated with FLX and 5HTP compared to controls and is significantly decreased in FLX + MDC. **B-C.** *Rab4a* and *Rab4b* mRNA expression remains unchanged with treatment. **D.** FLX significant decreases RhoA activity at 4 hours compared to lactogenic control with no other changes with treatment. FLX significantly decreases activity at 4 hours compared to baseline. All treatments reduce activity at 24-, 48-, and 72 hours compared to baseline. **E.** There was no change in Rac1 activity with treatment compared to control and no change with MDC treatment. Control was significantly reduced at 72 hours compared to baseline, FLX reduced activity at 4 hours and 48 hours compared to baseline, 5HTP reduced activity at 48 hours and 72 hours compared to baseline. Values are means and bars indicate SEM (n = 3 independent cell cultures). *p<0.05 **p<0.01 ***p<0.001 ****p<0.0001.

We assessed RhoA, Rab4a/b, and Rac1 as potential serotonylation target proteins in lactating HC11 cells. *RhoA* mRNA expression (Fig 4A) was significantly upregulated with FLX and 5HTP treatments (*p* = 0.0022 and *p* = 0.0100, respectively) compared to lactogenic control. FLX treatment in combination with the TG-inhibitor, MDC, reduced *RhoA* mRNA to lactogenic control levels (*p* = 0.0453); however, 5HTP in combination with MDC did not alter *RhoA* mRNA expression. *Rab4a* (Fig 4B) and *Rab4b* (Fig 4C) gene expression were not altered by treatment.

Subsequently, we examined RhoA activity (Fig 4D). RhoA activity was significantly decreased over time (*p*<0.0001), had a tendency for alteration by treatment (*p* = 0.0784), and had the presence of a treatment by time interaction (*p* = 0.0086). FLX significantly decreased RhoA activity at 4 hours (*p* = 0.0275) compared to lactogenic control, there were no other changes observed between treatments and lactogenic control. RhoA activity was consistently decreased at 24, 48, and 72 hours after lactogenic treatment compared to baseline levels across all time and treatments (*p*<0.0001), potentially due to degradation of Rho protein. Due to the position of 5HT binding to the switch 2 domain on RhoA, thereby producing an inability to hydrolyze the bound GTP, it is possible that RhoA is constitutively active until it is degraded by the proteasome [45]. The lactogenic control and 5HTP treatment had significantly decreased RhoA activity from 4 to 24 hours (*p* = 0.0008), with 5HTP remaining decreased until 48 hours (*p* = 0.0201). Finally, FLX decreased RhoA activity from 0 to 4 hours post-treatment (*p* = 0.0215).

Rac1 activity (Fig 4D) was also decreased over time (*p*<0.0001), with no changes occurring due to treatment (*p* = 0.1629), but there was a significant treatment by time interaction (*p* = 0.0473). Rac1 activity did not change due to either treatment when compared to control, with no change in activity when treated with MDC. The lactogenic control had significantly reduced Rac1activity at 72 hours (*p* = 0.0082) compared to baseline. FLX reduced Rac1 activity at 4 and 48 hours (*p* = 0.0126 and *p* = 0.0023, respectively) relative to baseline activity levels. Furthermore, 5HTP reduced Rac1 activity at 48 hours and 72 hours (*p* = 0.0098 and *p* = 0.0003, respectively) compared to baseline. Aside from FLX reducing Rac1 activity from 0 to 4 hours (*p* = 0.0126), there was no significant stepwise decline over time in activity due to any of the treatments.

## Discussion

PTHrP is synthesized and secreted from lactogenically differentiated mammary epithelial cells and in turn, 5HT can further stimulate this lactation-derived PTHrP synthesis, which is shown in this study and in previous studies [10, 22, 29]. However, 5HT’s regulation of PTHrP is not fully understood, with 5HT’s mechanism of action having been thought to be regulated through 5HT receptor 2B activation and altered DNA methylation of sonic hedgehog [10, 29]. Furthermore, during lactation there is a feedback loop between PTHrP and the calcium sensing receptor [46, 47]. When the calcium sensing receptor senses low calcium, it signals for PTHrP to be synthesized and secreted to liberate more calcium for the milk; conversely, when calcium is high, the receptor signals for less PTHrP to be synthesized. PTHrP is primarily present only during lactation, with the only other state seeing an upregulation in expression being breast cancer, catalyzed by the transforming growth factor β promoting bone metastases [48–50].

In this study we examined the molecular process of serotonylation as a potential mediator of increased PTHrP during lactation. To our knowledge, this is the first study examining serotonylation in normal mouse mammary epithelial cells that have been differentiated with lactogenic hormones. Our data suggests a novel potential mechanism for 5HT’s regulation of PTHrP, via serotonylation.

We used a common mouse mammary epithelial cell line (HC11) to study *in vitro* lactogenesis. Upon stimulation with lactogenic hormones (prolactin, insulin, and hydrocortisone), these cells undergo lactogenic differentiation characterized by the formation of mammospheres and upregulation of milk protein genes [43, 51, 52]. We demonstrated a constant expression of TG2 in HC11 cells, in both the undifferentiated and differentiated physiological states. Furthermore, we characterized TG activity, finding that as the lactational state of the mammary epithelial cells increases, the activity of TG increases. In the non-lactogenic state, the activity of TG decreased, however, it starts off higher than the lactogenic control does. Interestingly, TG2 is a calcium dependent enzyme [32]; therefore, due to the demand of calcium by the mammary gland for milk synthesis, it is possible that the influx of calcium is potentially allowing increased TG activity during lactogenesis [15].

We further went on to manipulate 5HT concentrations using two pharmacological mechanisms: treatment with FLX or 5HTP. We chose FLX and 5HTP, rather than 5HT due to the short half-life and rapid degradation of 5HT. The use of 5HTP or FLX has been used by our lab to increase 5HT in multiple studies [12, 22, 44, 53, 54]. We demonstrated that intracellular 5HT concentrations increase over time with both 5HTP and FLX treatment; however, at 48 hours, when these cells are fully differentiated, we no longer see an increase in 5HT concentration in 5HTP treated cells. This decrease is likely due to the degradation of 5HT by MAO. Since 5HTP bypasses the rate limiting step of 5HT synthesis, 5HTP is rapidly converted into 5HT, which is then rapidly degraded by MAOs, particularly MAO-A [4, 5, 55]. Extracellular 5HT is taken into the cell by SERT and is cleared via MAO in the intracellular space [56]. Therefore, treatment with FLX, which blocks SERT, fails to upregulate the degradation of 5HT by MAO-A, resulting in increased intracellular 5HT.

Both 5HTP and FLX increase *de novo* synthesis of 5HT. This is catalyzed by the upregulation of TPH1 gene expression; however, only FLX increased SERT expression, suggesting only FLX inhibits SERT. Since the mechanism of action FLX’s action is to inhibit SERT, the increase in gene expression is necessary as a method to compensate for the inhibition. Many studies have examined SSRIs and their corresponding action on SERT protein and gene expression and have found mixed results, often attributed to the duration, type of drug, and dose administered [57–61].

As expected, the resulting increases in intracellular 5HT concentration with either 5HTP or FLX treatment sufficiently upregulated *Pthlh* mRNA expression, recapitulating previous data [10, 22, 44]. Interestingly *Pthlh* mRNA remains at a similar expression level in the non-lactogenic cells compared to the lactogenic control cells, however the expression level significantly increases with FLX or 5HTP treatment in the non-lactogenic controls (S1 Fig), suggesting a serotonin effect on PTHrP in both physiological states, which warrants future research.

We then determined whether serotonylation is involved in the induction of PTHrP by using the TG inhibitor, MDC, to reduce serotonylation. Many studies have attributed MDC’s inhibitory effects on signaling pathways to the process of serotonylation [35, 37, 62]. Consistent with our hypothesis, when we combined 5HTP or FLX treatment with MDC, *Pthlh* expression was decreased and restored to lactogenic control levels, suggesting a TG-dependent serotonylation action. This is one mechanism likely involved in 5HT’s regulation of PTHrP synthesis.

PTHrP and the parathyroid hormone (PTH) bind to the same PTH-receptor, and PTHrP activity has commonly been measured using cAMP concentration, which is downstream of the PTH-receptor [22, 63, 64]. We determined that 5HTP treatment significantly increased cAMP concentrations, while FLX treatment resulted in a significant decreased in cAMP concentrations compared to lactogenic control under lactogenic conditions. Only 5HTP increased the PTH-receptor (Pthr1) gene expression at 48h in the lactogenically differentiated cells (data not shown). This upregulation of the receptor, as well as the increase in cAMP at 48h may be a result of the dynamics of 5HT turnover. Since 5HTP bypasses the rate limiting step in 5HT synthesis, it is rapidly converted to 5HT compared to FLX which relies on the upregulation of *de novo* synthesis of 5HT. Furthermore, MAO-A is upregulated with 5HTP, lending support to 5HTP increasing turnover of 5HT. These dynamics of 5HT concentrations have been shown in our lab, as well in these experiments, where 5HTP has effects within hours compared to days with FLX [22, 44].

The decrease in cAMP with FLX treatment under lactogenic conditions is in contrast to previous results in our lab where we determined that FLX exposure in rodents increased mammary gland cAMP concentrations on day 10 of lactation after approximately 14 days of treatment with FLX [22]. We speculate that both duration and timing of treatment (acute vs. chronic) with these compounds, FLX and 5HTP, can result in different effects on the mammary gland and other tissues, which have been previously demonstrated [53, 65]. Interestingly, in the human breast cancer cell line, MDA-MB-231, 5HT decreased cAMP concentrations and resulted in a switch from growth inhibition to growth stimulation, due to the change in cAMP dynamics [66]. Furthermore, in lymphocytes, FLX has been shown to have dual effects where it can either increase or decrease cAMP concentrations depending on whether it is growth-promoting or inhibitory, resulting in modulation of the immune response [67, 68]. Therefore, it is possible that our observed decrease in cAMP may be due to pharmacological properties of FLX, rather than activity of PTHrP.

While the non-lactogenic control had increased cAMP compared to the lactogenic control, it is important to note the media has insulin and FBS. Interestingly, we do find a similar result in the non-lactogenic cells treated with FLX or 5HTP as we do in the lactogenic (S1 Fig) where 5HTP significantly increases cAMP while FLX moderately decreased cAMP. This suggests that there may a serotonin-mediated effect in both the lactogenic and non-lactogenic cells. Furthermore, most 5HT receptors are G-protein coupled receptors, which can use cAMP as a secondary messenger upon binding of 5HT [28]. Intracellular serotonin may be released from the cell where it binds in an autocrine fashion to one of the 5HT receptors, producing a cAMP response in the non-lactogenic controls.

MDC is a commonly used TG inhibitor used to manipulate serotonylation. In our study, MDC did not completely abolish PTHrP expression or reduce cAMP concentrations. It is possible these results are due to the low concentration of MDC used in this study (25μM). The concentration of MDC used in this experiment was substantially lower than many serotonylation studies which have ranged from 200μM up to 5mM of MDC [38, 45, 62, 69]. We chose the lower concentration of MDC due to chronic duration of treatment (48+ hours), compared to the acute duration (1–8 hours) of the studies using higher concentrations of MDC. However, studies have shown effects at lower concentrations; one study observed that 20μM MDC treatment overnight resulted in a significant decrease in 5HT mitogenesis of distal primary bovine arterial smooth muscle cells, though higher doses (up to 200μM) resulted in larger decreases [62]. In an additional study it was determined that a 25μM MDC treatment for 3 hours in L6 rat muscle cells results in significant decreases in 5HT-induced effects on GLUT4 translocation, glucose uptake, and glycogen content [35]. We therefore concluded a lower dose would sufficiently inhibit serotonylation; however, it is likely we would have seen further reductions in PTHrP and a reduction in cAMP concentrations if we used a higher concentration of MDC. Furthermore, PTHrP is tightly regulated during lactation and serotonylation may only be one mechanism involved. Therefore, using an even higher dose of MDC may not completely abolish PTHrP expression due to the compensatory mechanisms through the 5HT receptor 2B activation and calcium sensing receptor [10, 47].

Serotonylation has been established to occur via transamidation mediated by the TG enzyme. TG is implicated in many other cellular processes, including monoaminylation (e.g. serotonylation, histaminylation, dopaminylation, and norepinephrinylation) and protein-protein crosslinking involved in extracellular matrix proteins, growth factor activity, integrin activity, oxidative stress/inflammation, and EGF/EGFR signaling in epithelial cancer cells [70–73]. Therefore, we speculate that by increasing 5HT concentrations, we are increasing serotonylation through increased substrate (5HT) availability. We then characterized TG activity, where we illustrated TG activity with respect to time and treatment. As expected, treatment with MDC resulted in no changes in TG activity. MDC is a small-molecule inhibitor that acts as an alternative substrate, exploiting the protein-crosslinking activity of TG [74]. It has been shown that MDC will outcompete the donor substrate (5HT), thereby reducing serotonylation [74, 75].

To determine the downstream signaling targets of serotonylation due to FLX or 5HTP treatment in lactogenic mammary epithelial cells we investigated G-proteins. G-proteins have emerged as the most common serotonylation target. Herein, we probed a variety of G-proteins including: RhoA, Rab4a/b, and Rac1. When G-proteins such as the Rho and Rab family are serotonylated, they lose the intrinsic ability to hydrolyze GTP to GDP, thus rendering them constitutively active until proteasomal degradation [31, 32, 39, 76]. A serotonylation study in pulmonary hypertension in smooth muscle cells has shown RhoA protein to be significantly decreased at 24, 48, and 72 hours post 5HT stimulation, with decreased protein being attributed to the degradation feedback to eliminate activity [45]. Consistent with this study, when examining RhoA activity, we found a consistent significant decrease, whereas Rac1 activity remained relatively constant from 4 to 48 hours. This may suggest Rac1 undergoes minimal degradation, suggesting it is unlikely to be serotonylated.

We speculate that RhoA is potentially a serotonylation target in lactating mammary epithelial cells, though we have not directly confirmed whether serotonylation of RhoA is involved in the regulation of PTHrP. However, studies have shown RhoA’s involvement in increased PTHrP expression, though the exact mechanism still needs to be elucidated [77, 78]. Together, this supports our hypothesis that serotonylated RhoA may regulate PTHrP induction. Although we do not see much change in RhoA activity with treatment, the step-wise decrease in Rho activity coupled with the increase in gene transcription, suggests RhoA may be degraded for inactivation and transcription increases to replenish the degraded RhoA protein. Furthermore, PTHrP has been shown to increase RhoA; therefore, the increase in RhoA transcription may be a result of increased PTHrP [79]. Together, PTHrP and RhoA may result in a positive feedback loop where serotonylated RhoA increases PTHrP expression and increased PTHrP results in increased RhoA expression.

To our knowledge, this study is the first to examine serotonylation in normal mammary epithelial cells under lactogenic conditions. We conclude that 5HT may regulate PTHrP in part through the novel mechanism of serotonylation. PTHrP synthesis during lactation is tightly regulated through many mechanisms in the mammary epithelial cells potentially including 5HT receptor 2b activation, altered DNA methylation of sonic hedgehog, calcium sensing receptor, and now serotonylation.

## Supporting information

S1 Fig Non-lactogenic control cells. **A.** Intracellular 5HT concentration is significantly increased in FLX treated non-lactogenic HC11 cells. **B.** Transglutaminase activity is significantly decreased over time in all treatments, except the non-lactogenic control cells treated with MDC. MDC significantly increased TG activity at 24 and 48 hours compared to non-lactogenic control. 5HTP decreased TG activity at 24 and 48 hours while FLX decreased TG activity only at 48 hours compared to non-lactogenic control cells. **C.** cAMP concentration was significantly upregulated at 24, 48, and 72 hours in 5HTP treated cells, whereas FLX decreased cAMP at 48 and 72 hours compared to non-lactogenic control cells. **D-G.** Gene expression after 48 hours of treatment. **D.** Pthlh mRNA was significantly increased with FLX or 5HTP and was significantly reduced to non-lactogenic control levels with treatment with MDC. **E.** Mao-a mRNA was significantly increased with 5HTP. **F.** Sert mRNA was significantly increased with 5HTP. **G.** Tph1 mRNA was significantly decreased with FLX or 5HTP.
(TIFF)

S1 Raw images (TIF)

## Abbreviations

5HT: serotonin
5HTP: 5-hydroxytryptophan
cAMP: cyclic adenosine monophosphate
FLX: fluoxetine
MAO: monoamine oxidase
MDC: (mono)dansylcadaverine
PTHrP: parathyroid hormone related protein
SERT: serotonin reuptake transporter
SSRI: selective serotonin reuptake transporter
TG: transglutaminase
TPH: tryptophan hydroxylase
