# Supplementary figures and images for: Serotonin stimulated parathyroid hormone related protein induction in the mammary epithelia by transglutaminase-dependent serotonylation
Source: PLoS One. 2020 Oct 23;15(10):e0241192. doi: 10.1371/journal.pone.0241192 (PMC7584195; doi:10.1371/journal.pone.0241192)

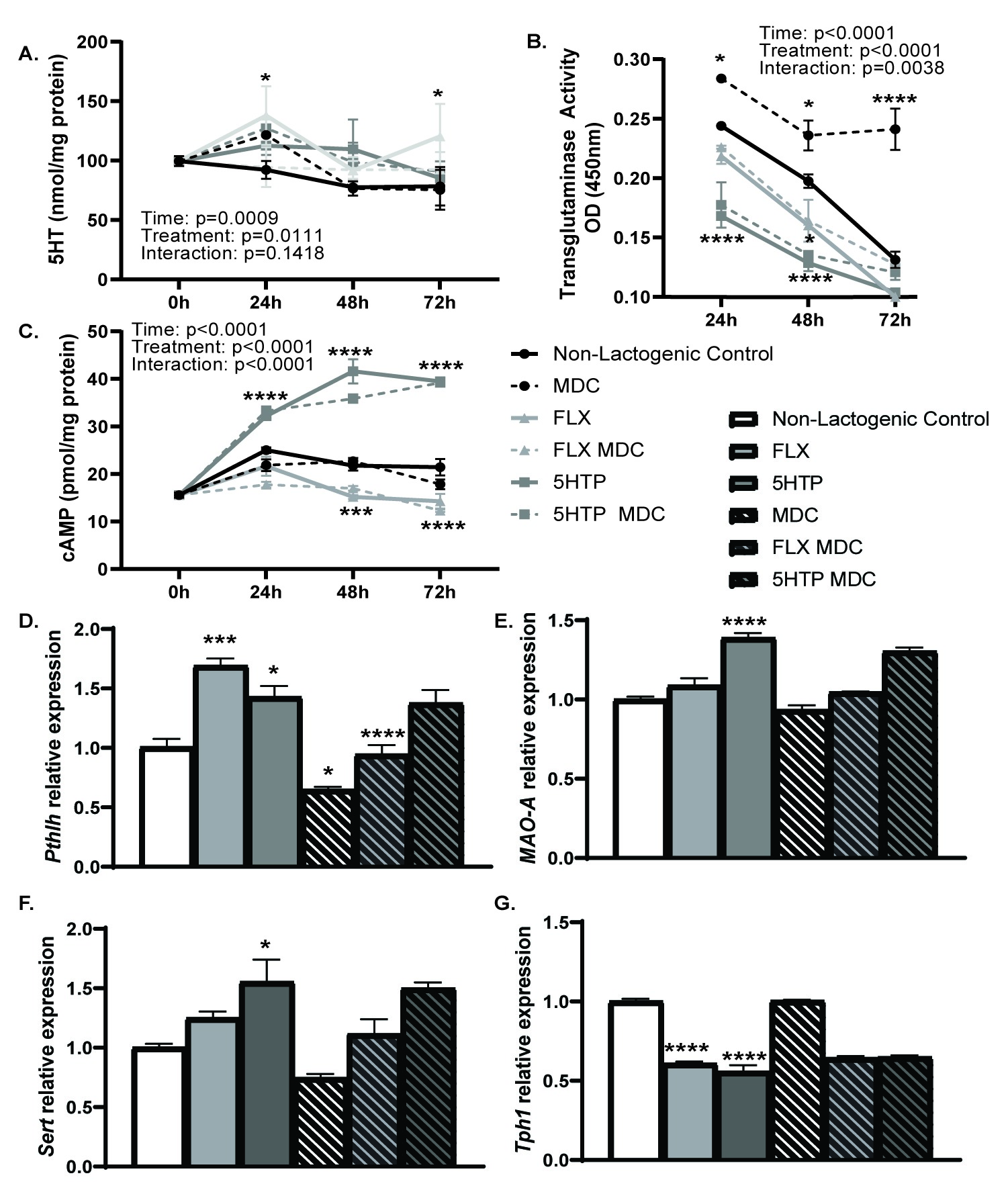

Supplement: S1 Fig — A. Intracellular 5HT concentration is significantly increased in FLX treated non-lactogenic HC11 cells. B. Transglutaminase activity is significantly decreased over time in all treatments, except the non-lactogenic control cells treated with MDC. MDC significantly increased TG activity at 24 and 48 hours compared to non-lactogenic control. 5HTP decreased TG activity at 24 and 48 hours while FLX decreased TG activity only at 48 hours compared to non-lactogenic control cells. C. cAMP concentration was significantly upregulated at 24, 48, and 72 hours in 5HTP treated cells, whereas FLX decreased cAMP at 48 and 72 hours compared to non-lactogenic control cells. D-G. Gene expression after 48 hours of treatment. D. Pthlh mRNA was significantly increased with FLX or 5HTP and was significantly reduced to non-lactogenic control levels with treatment with MDC. E. Mao-a mRNA was significantly increased with 5HTP. F. Sert mRNA was significantly increased with 5HTP. G. Tph1 mRNA was significantly decreased with FLX or 5HTP. (TIFF) [file pone.0241192.s001.tiff]

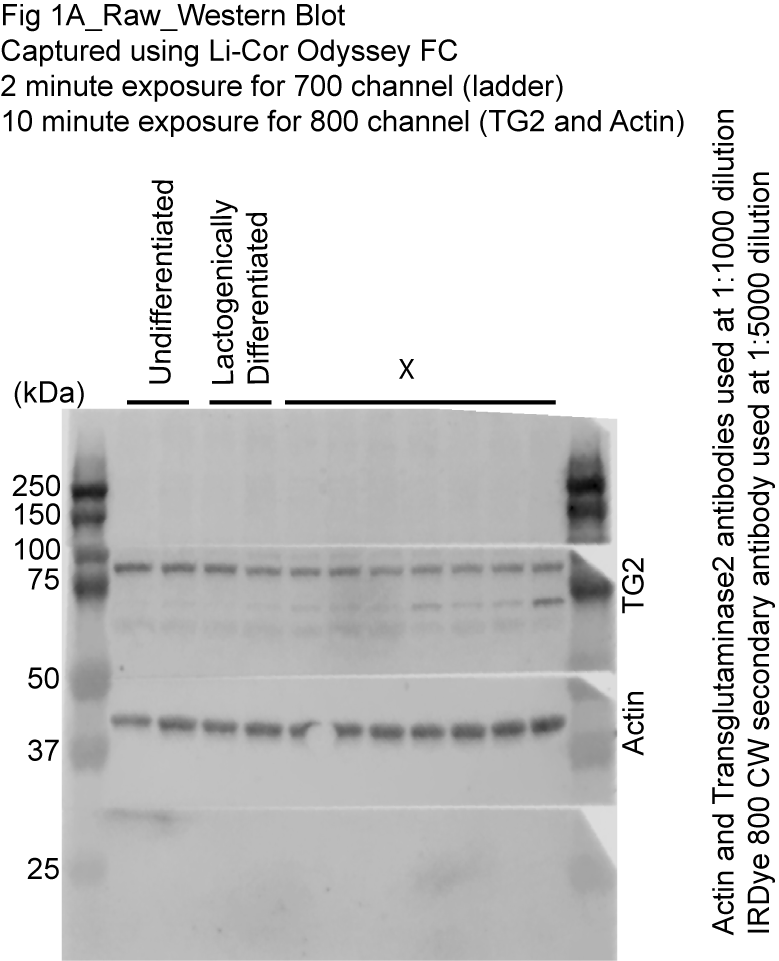

Supplement: S1 Raw images — (TIF) [file pone.0241192.s002.tif]
